# Risk Factors for Enteric Pathogen Exposure among Children in Black Belt Region of Alabama, USA

**DOI:** 10.3201/eid2912.230780

**Published:** 2023-12

**Authors:** Drew Capone, Toheedat Bakare, Troy Barker, Amy Hutson Chatham, Ryan Clark, Lauren Copperthwaite, Abeoseh Flemister, Riley Geason, Emery Hoos, Elizabeth Kim, Alka Manoj, Sam Pomper, Christina Samodal, Simrill Smith, Claudette Poole, Joe Brown

**Affiliations:** Indiana University, Bloomington, Indiana, USA (D. Capone);; University of North Carolina at Chapel Hill, Chapel Hill, North Carolina, USA (T. Bakare, T. Barker, R. Clark, L. Copperthwaite, A. Flemister, R. Geason, E. Hoos, E. Kim, A. Manoj, S. Pomper, C. Samodal, J. Brown);; University of Alabama at Birmingham, Birmingham, Alabama, USA (A. Hutson Chatham, C. Poole);; Georgia Institute of Technology, Atlanta, Georgia, USA (S. Smith)

**Keywords:** enteric infections, Black Belt, enteric pathogens, well water, straight pipe, sanitation, Alabama, United States

## Abstract

We collected stool from school-age children from 352 households living in the Black Belt region of Alabama, USA, where sanitation infrastructure is lacking. We used quantitative reverse transcription PCR to measure key pathogens in stool that may be associated with water and sanitation, as an indicator of exposure. We detected genes associated with >1 targets in 26% of specimens, most frequently *Clostridioides difficile* (6.6%), atypical enteropathogenic *Escherichia coli* (6.1%), and enteroaggregative *E. coli* (3.9%). We used generalized estimating equations to assess reported risk factors for detecting >1 pathogen in stool. We found no association between lack of sanitation and pathogen detection (adjusted risk ratio 0.95 [95% CI 0.55–1.7]) compared with specimens from children served by sewerage. However, we did observe an increased risk for pathogen detection among children living in homes with well water (adjusted risk ratio 1.7 [95% CI 1.1–2.5]) over those reporting water utility service.

Outside cities and towns served by conventional sewerage, many residents in the rural Black Belt region of Alabama, USA, have failing or inadequate sanitation infrastructure (*1*,*2*). This region was named after its rich black soils, which are typically high in clay content, limiting subsurface infiltration (*3*) and leading to surface discharge of domestic wastewater. Compounding those challenges is a high rate of poverty; 9 of the 10 poorest counties in Alabama are in the Black Belt region (*2*,*4*). Because common alternatives to septic systems are unaffordable (*5*,*6*), many residents use failing systems or lack systems altogether (*7*,*8*). Straight piping (i.e., direct discharge of untreated fecal wastes to the environment) of domestic wastewater is common (*7*).

When human fecal wastes are not safely managed, they may be transported to the environment through well-understood fecal–oral pathways (i.e., drinking water, soils, flies, food, fomites, and hands) (*9*,*10*). For households reliant on straight pipe discharge of wastewater, direct exposure to this waste may be more likely than for households served by a septic system (*8*). Those same households and their communities may also suffer from exposures further downstream. Inadequate treatment of fecal wastes can result in enteric pathogen transport through soil into groundwater and exposure through drinking water (e.g., well water) (*11*,*12*). Other exposures may include fecally contaminated soils (*13*), flies that feed on and reproduce in human feces (*14*,*15*), and contaminated food (*10*). Such exposures can result in infection with enteric pathogens, which is a necessary precondition for diarrheal disease and other sequelae, including environmental enteric dysfunction (*16*), growth deficits (*17*), cognitive impairment (*18*), and negative effects on the immune system (*19*).

Poor sanitation and persistent exposure to fecal wastes, particularly in the context of a state and nation with ample resources to address the issue (*20*), represents a public policy failure (*7*,*21*) affecting human health, dignity, and quality of life. Although the evidence base for public investment in sanitation on health grounds has a long history (*22*), the health burden attributable to poor rural sanitation in the United States remains poorly characterized, constraining the case for action. To determine the potential roles of rural sanitation improvements or other interventions in controlling disease transmission, a useful first step is estimating prevalence of enteric infections and identifying risk factors associated with them. Because of documented poor sanitation conditions in Alabama’s Black Belt region (*5*,*7*,*8*) and the associated potential persistence of endemic enteric infection (*23*–*25*), we conducted a cross-sectional study to assess the prevalence of stool-based enteric pathogen detection in children using molecular methods, as an indicator of previous exposure. We further sought to identify potential household-level environmental risk factors for exposure to those pathogens to understand the potential role of infrastructure in protecting public health in this underserved region.

## Methods

### Study Site and Participants

This study was nested within a larger cross-sectional helminth surveillance study in rural Alabama (*26*). Participants were children 2–18 years of age living in 3 counties in the Black Belt (Lowndes, Wilcox, and Perry Counties). All children included in a household were requested to participate. The study used principles of community-based participatory research to work with stakeholders in co-creation of the project (*27*). Several meetings and focus groups were held with community partners and study collaborators to help guide study protocols, recruitment methods and materials, and participant enrollment. Participants were enrolled during January 2019–December 2021 (*26*).

We provided participants with an at-home stool collection kit. For 3 separate bowel movements, participants filled and returned 1 50-mL collection tube containing 15 mL of zinc polyvinyl alcohol (Zn-PVA) (*28*) with ≈15 g of stool and another tube containing 15 mL of 10% formalin with an additional 15 g of stool (Parapak; Meridian Bioscience). Participants received $150 on a prepaid debit card for their participation. In addition, participants completed a brief paper questionnaire regarding their demographic characteristics, household sanitation infrastructure, and potential exposures. Before March 2020, the questionnaire was completed at the time of enrollment; after March 2020, the questionnaire was completed by the participant at home and mailed to the study team.

### Molecular Analysis

During January 2019–November 2020, specimens were shipped at ambient conditions to Georgia Institute of Technology (Atlanta, GA, USA); during December 2020–December 2021, specimens were shipped at ambient conditions to the University of North Carolina at Chapel Hill (Chapel Hill, NC, USA). Upon receipt, we visually screened specimens for indicators of nonhuman origin (i.e., animal hair, dirt, color, and atypical morphology), homogenized them with sterile inoculating loops (VWR; Radnor) and stored them at 4°C for further analysis. By using the QIAamp 96 Virus QIAcube HT Kit (QIAGEN), which included a pretreatment step with Precellys SK38 bead beating tubes (Bertin Technologies) (*29*–*31*), we extracted total nucleic acids from ≈150 mg of the stool Zn-PVA mixture. We typically extracted specimens within 1–4 weeks of receipt (median 15 days, interquartile range 8–28 days). We analyzed extracts from specimens suspected to potentially be from nonhuman sources by using dPCR (QIAcuity 4; QIAGEN) for human mitochondrial DNA, using a previously validated method that has high sensitivity and specificity for human feces (*32*). Among children who submitted >1 stool specimen, we randomly selected a single replicate for extraction. We randomly selected ≈5% of stools for duplicate extraction and another 3% for extraction from multiple replicates. We included >1 extraction-negative control (*33*) during each day of extractions. We spiked specimens with 10^7^ copies of bacteriophage MS2 and 10^6^ gene copies of synthetic DNA (IDT) as extraction-positive controls. We stored extracts at –80°C until analysis.

We measured 30 enteric pathogens in specimens by using a custom TaqMan Array Card (TAC) on a Quantstudio 7 Flex (ThermoFisher) at the University of North Carolina at Chapel Hill, according to the methods described by Liu et al. (*34*). Targets were *Acanthamoeba* spp., adenovirus 40/41, astrovirus, *Balantidium coli*, *Blastocystis* spp., *Cystoisospora belli*, *Cyclospora cayetanensi*, *Campylobacter jejuni* or *C.* *coli*, *Clostridioides difficile*, *Cryptosporidium* spp., *Enterocytozoon bieneusi*, *Escherichia coli* O157:H7, *Encephalitozoon intestinalis*, *Entamoeba hystolytica*, *Entamoeba* spp., enteroaggregative *E. coli*, enteropathogenic *E. coli*, enterotoxigenic *E. coli*, *Giardia* spp., *Helicobacter pylori*, hepatitis A virus, *Shigella* spp. or enteroinvasive *E. coli*, norovirus, *Plesiomonas shigelloides*, rotavirus, *Salmonella* spp., sapovirus, SARS-CoV-2, Shiga toxin–producing *E. coli*, and *Yersinia enterocolitica*. We prepared the TAC by combining 40 µL of template with 60 µL of AgPath-ID One-Step RT-PCR Reagents (Applied Biosystems). We evaluated TAC performance by using an 8-fold dilution series (10^9^–10^2^ gene copies/reaction) of an engineered combined positive control developed by using methods from Kodani and Winchell 2012 (*35*). We used 2 plasmids (GeneArt), including 1 specifically for DNA targets. We linearized the other with a BshT1 restriction enzyme (ThermoFisher) and transcribed it (MEGAscript T7 Transcription Kit and MEGAclear Transcription Clean-Up Kit, both from ThermoFisher) to generate RNA control material, which we quantified by using a Qubit RNA HS Assay Kit on Qubit 4 Fluorometer (ThermoFisher). The linearity and efficiency for 28 of the 30 targets were within normative standards (linearity 0.97–1.0, efficiency 87%–102%) (Appendix Tables 1–3, Figure 1). The assays for hepatitis A virus and adenovirus 40/41 did not perform well, and we excluded them from our analysis.

Each day of TAC analysis, we included >1 positive and negative control (either an extraction-negative control or a PCR-negative control). We determined quantification cycle values through manual thresholding and included comparison of each specimen’s fluorescent signal against the daily negative and positive controls (Appendix Figure 1). We categorized any target that amplified past a quantification cycle of 35 as negative to reduce the potential for false positives (*34*). To examine the effect of our preservation medium on the probability of detecting our targets of interest, we measured recovery of *Giardia duodenalis* and *Shigella sonnei* from stool by using different preservative conditions over a period of 8 weeks (Appendix).

### Data Analysis

To perform Poisson regression, we used generalized estimating equations with robust SEs that accounted for clustering among children living in the same household. This method estimated unadjusted and adjusted risk ratios with 95% CIs. We created a directed acyclic graph on the basis of the variables included in the questionnaire where independent variables were biologically plausible predictors of the dependent variable, which was the detection of nucleic acids from >1 enteric pathogen in stool (Appendix Figure 2). Independent variables that met this criterion were the household’s sanitation infrastructure, whether the household paid a water bill (i.e., a proxy measure indicating a connection to a water utility), reported raw sewage in the home, and the child’s screen time, sex, history of international travel, and age. We generated adjusted estimates from a single model that included all independent variables. We used multiple imputation by chained equations (MICE package in R [*36*]) with 10 multiple imputations and the predictive mean matching method to account for missing data in the generalized estimating equations model.

### Ethics Considerations

We obtained written informed consent from each participant’s legal guardian and assent from children >7 years of age. The study protocol was approved by the Institutional Review Boards of the University of Alabama at Birmingham (approval no. 300002219), Georgia Institute of Technology (approval no. H19021), and the University of North Carolina at Chapel Hill (approval no. 20-3212).

## Results

### Questionnaire

We enrolled 488 children from Wilcox (237 participants from 181 households), Lowndes (101 participants from 50 households), and Perry Counties (86 participants from 55 households) (Table 1). Most children identified as Black or African American (91% [444/488]); few identified as White (2% [9/488]), preferred not to answer (1% [6/488]), identified as Black and White (<1% [2/488]), or were unsure (<1% [2/488]). Almost half of households (47% [164/352 households) enrolled multiple children (63% [306/488] participants). The median age of enrolled children was 11 years (range 2–18 years, interquartile range 8–14 years). A septic tank system was the most reported sanitation infrastructure (42% [207/488] of participants, 39% 137/352 of households), followed by a sewer connection (23% [111/488] of participants, 20% [72/352] of households,), whereas 11% (56/488) of respondents (11% [39/352] of households) reported straight piping wastewater onto their property. Few participants reported not paying a water bill (14% [67/488] of participants, 14% [48/352] of households), an indicator of household-based well water usage. As a proxy for time spent indoors, participants most often reported >4 hours of screen time per day (42% [203/488]), followed by 2–4 hours (37% [182/488]) and <2 hours (15% [72/488]).

**Table 1:** Demographic characteristics of 488 children and water infrastructure summary based on self-administered surveys conducted in Lowndes, Wilcox, and Perry Counties, Alabama, USA, January 2019–December 2021*

Variable and response	Value
Race	
Black or African American	444 (91)
White	9 (1.8)
Prefer not to answer	6 (1.2)
Black and White	2 (0.4)
Unsure	2 (0.4)
No response	25 (5.1)
Ethnicity	
Not Hispanic or Latino	407 (83)
Prefer not to answer	16 (3.3)
Hispanic or Latino	7 (1.4)
Unknown	6 (1.2)
No response	52 (11)
County	
Wilcox	237 (56)
Lowndes	101 (21)
Perry	86 (17)
No response	66 (14)
Household receives water bill	
Yes	385 (79)
No	67 (14)
Don’t know	6 (1.2)
No response	430 (6.1)
Household sanitation	
Septic tank	207 (42)
Sewer connection	111 (23)
Don’t know	80 (16)
Straight pipe	56 (11)
Cesspit	2 (0.4)
Other	1 (0.1)
No response	31 (6.3)
Raw sewage in yard or home in past year	
No	400 (82)
Yes	38 (7.8)
No response	50 (10)
History of international travel in past year	
No	448 (92)
Yes	13 (2.7)
No response	27 (5.5)
Sex	
M	236 (48)
F	229 (47)
No response	23 (4.7)
Daily screen time, h	
<2	72 (15)
2–4	182 (37)
>4	203 (42)
No response	31 (6.4)
Age, y	
Mean (SD)	11 (4.1)
Median (interquartile range)	11 (8–14)
Range	2–18
No response	37 (17.6)
Ever treated for an intestinal parasite	
No	418 (86)
Don’t know	45 (9.2)
Yes	12 (2.5)
No response	13 (2.7)

*Values are no. (%) except as indicated.

### Reverse Transcription Quantitative PCR

We detected target-specific nucleic acids from >1 pathogen in 26% (127/488) of children’s stool specimens (Table 2), most frequently *C. difficile* (6.6% [32/488]), atypical enteropathogenic *E. coli* (6.1% [30/488]), and enteroaggregative *E. coli* (3.9% [19/488]). We detected each viral, protozoan, fungal, and algae targets in <1.0% of specimens except for *Blastocystis* (3.7% [18/488]) and norovirus genotype group I or II (1.4% [7/488]). We observed perfect agreement in target detection among 26 specimens analyzed in duplicate (same child, same bowel movement) and 80% (12/15) agreement in pathogen detection among replicates (same child, different bowel movement). We did not observe contamination among extraction-negative controls (n = 19) and PCR-negative controls (n = 2), and our PCR-positive controls (n = 30) exhibited the expected amplification for all targets except hepatitis A virus and adenovirus 40/41.

**Table 2:** Prevalence of enteric pathogens in stool specimens of children in a study conducted in Lowndes, Wilcox, and Perry Counties, Alabama, USA, January 2019–December 2021*

Type and pathogen	Prevalence, no. (%)
Any	
>1 pathogen gene detected	127 (26)
Bacteria	
* Clostridioides difficile*	32 (6.6)
EPEC (atypical)	30 (6.1)
EAEC	19 (3.9)
* Helicobacter pylori*	11 (2.3)
EPEC (typical)	7 (1.4)
* Yersinia enterocolitica*	5 (1.0)
*E. coli* O157:H7	4 (0.8)
* Plesiomonas shigelloides*	2 (0.4)
ETEC	2 (0.4)
*Shigella* or EIEC	1 (0.2)
* Salmonella*	1 (0.2)
STEC	1 (0.2)
* Campylobacter jejuni or coli*	0
Fungus/algae	
* Blastocystis*	18 (3.7)
* Enterocytozoon bieneusi*	0
* Encephalitozoon intestinalis*	0
Protozoa	
* Balantidium coli*	3 (0.6)
* Acanthamoeba*	2 (0.4)
*Giardia* spp.	2 (0.4)
* Entamoeba hystolytica*	1 (0.2)
* Cystoisospora belli*	0
* Cyclospora cayetanensi*	0
* Cryptosporidium*	0
* Entamoeba*	0
Virus	
Norovirus GI or GII	7 (1.4)
SARS-CoV-2	3 (0.6)
Rotavirus	2 (0.4)
Sapovirus	2 (0.4)
Astrovirus	1 (0.2)

*EAEC, enteroaggregative *Escherichia coli*; EIEC, enteroinvasive *E. coli*; EPEC, enteropathogenic *E. coli*; ETEC, enterotoxigenic *E. coli*; GI/GII, genotype group I and II; STEC, Shiga toxin–producing *E. coli*.

### Risk Factor Analysis

We found no association between pathogen detection in samples from participants who reported using a straight pipe or septic tank compared with those served by a sewer connection (Table 3). The only statistically significant association we observed, according to the conventional definition of significance (*37*), was that participants from households that did not pay a water bill (a proxy for well water consumption) had a greater risk (adjusted risk ratio [aRR] 1.7 [95% CI 1.1–2.5]) of detection of >1 pathogen than did participants from households that reported paying a water bill. Although not meeting conventional definitions of statistical significance (*37*), the point estimates for 2–4 hours of screen time (aRR 0.79 [95% CI 0.51–1.2]) and >4 hours of screen time (aRR 0.73 [95% CI 0.47–1.1]) suggest that time spent indoors could be protective against enteric pathogen detection, although this observation should be interpreted with caution. We found minor differences in the regression results using only complete cases (n = 341) compared with the model that used MICE (Appendix Table 4); not paying a water bill was associated with increased risk for detecting >1 pathogen targets (aRR 1.8 [95% CI 1.3–2.6]), and >4 hours of reported daily screen time had a greater protective effect at the margin of significance (aRR 0.64 [95% CI 0.41–1.0]).

**Table 3:** Risk factors for detection of >1 enteric pathogen in stool specimens of children in a study conducted in Lowndes, Wilcox, and Perry Counties, Alabama, USA, January 2019–December 2021*

Variable	Reference	Exposure	RR (95% CI)	aRR (95% CI)
Pay a water bill	Yes	No	1.8 (1.2–2.5)	1.7 (1.1–2.5)
Sanitation	Sewer connection	Cesspit	NA	NA
		Other	3.4 (0.57–20)	5.2 (0.88–30)
		Septic tank	0.89 (0.61–1.3)	0.95 (0.64–1.4)
		Straight pipe	0.95 (0.55–1.6)	0.95 (0.55–1.7)
Child’s screen time	<2 h	2–4 h	0.74 (0.48–1.1)	0.79 (0.51–1.2)
		>4 h	0.74 (0.48–1.1)	0.73 (0.47–1.1)
Child’s sex	Male	Female	0.89 (0.65–1.2)	0.89 (0.65–1.2)
International travel in past year	No	Yes	0.89 (0.32–2.5)	0.93 (0.34–2.5)
Raw sewage in home or yard in past year	No	Yes	1.1 (0.68–1.9)	1.1 (0.66–2.0)
Child’s age	<5 y	5–10 y	0.71 (0.40–1.3)	0.76 (0.41–1.4)
		>10 y	0.82 (0.47–1.4)	0.90 (0.49–1.6)

*Unadjusted RRs are from bivariate models, whereas aRRs are from full model including all covariates. aRR, adjusted risk ratio; RR, risk ratio.

### Human Stool Specimen Confirmation

One stool specimen was flagged by technicians as potentially nonhuman because of atypical morphology. In addition, we prospectively selected 51 additional specimens for screening to determine origin. All specimens were positive for human mitochondrial DNA at concentrations indicating human origin (*32*). The median concentration was 10^3.3^ gene copies human mitochondrial DNA per nanogram of double-stranded DNA (range 10^1.2^–10^4.7^ gene copies/nanogram double-stranded DNA).

### Zn-PVA Validation

The concentration of *Giardia* DNA we recovered from Zn-PVA decreased by 0.034 log_10_/day at ambient conditions and by 0.0037 log_10_/day in Zn-PVA at 4°C. The concentration of *Shigella* DNA we recovered from Zn-PVA decreased at ambient conditions by 0.030 log_10_/day and by 0.0085 log_10_/day in Zn-PVA at 4°C (Appendix Table 5, Figure 3).

## Discussion

We detected various enteric pathogens in stool specimens from children living in the Black Belt of Alabama. Straight pipe sanitation (direct discharge of fecal wastes into the environment near households) was not associated with increased risk for stool pathogen detection compared with conventional sewerage. However, our finding that well water consumption was associated with an increased risk for enteric pathogen detection implicates poor sanitation in this geographic area as a possible contributor to groundwater contamination. Soils that are high in clay content undergo shrinking as they desiccate and swelling as they moisten (*3*). Those conditions may lead to fecal waste transport from failing septic tanks and straight pipe discharges through soils to the water table (*3**,**38*), resulting in exposures through drinking water. Previous work in the Black Belt observed an increased concentration of fecal contamination in well water compared with piped municipal water. In a cross-sectional study of randomly selected households in Hale County (bordering Perry County in the Black Belt), 20% of private wells were positive for fecal coliforms, compared with 8% of public water system specimens (*12*). Other studies from the region have reported fecal contamination of water supplies, possibly linked to widespread sanitation deficits (*11*,*39*,*40*).

We used detection of pathogens in stool as a proxy for carriage and as an unambiguous indicator of previous exposure (*41*), a suitable measure given the role of water and sanitation infrastructure in limiting exposures to many of the pathogens we assessed. It is important to note that detecting a pathogen in stool does not necessarily indicate the person experienced symptomatic or asymptomatic infection. For example, detecting *C. difficile* by PCR does not guarantee the presence of *C. difficile* toxin, and infection without the presence of this toxin may not result in diarrheal disease (*42*). Further, the relationship between carriage, infection, and disease is highly host- and pathogen-specific (*43*). Evidence from an international multisite study on the etiology of diarrhea in children posited that the detection of enteroaggregative *E. coli* at low concentrations in stool appeared to be protective against diarrhea, whereas detection of pathogens such as *Helicobacter pylori*, *Shigella*, and norovirus were strongly associated with diarrhea (*43*). Important microbiome-mediated interactions between and among pathogens are possible, and host responses can vary.

Compared with data for children in low- and middle-income countries, the 26% combined prevalence of enteric pathogens we observed is dramatically lower than what has been previously reported (*29*,*43*). Few studies have screened populations for multiple enteric pathogens in high-income countries outside of clinical settings or from asymptomatic populations. A study of 438 children in daycare centers in Uppsala, Sweden, from 2016 tested for 21 different enteric pathogens using PCR and detected >1 pathogen in stool specimens from 3.7% of children (*44*). The pathogens they detected most frequently were *C. difficile* (2.5%), adenovirus 40/41 (1.6%), *Campylobacter* (0.7%), and norovirus (0.7%) (Appendix Table 6). A 2001 study of 1,091 asymptomatic children and adults in Australia assessed 28 pathogens and detected >1 pathogen in 2.6% of stool specimens, including *Giardia* (1.6%), *Salmonella* (0.4%), *Cryptosporidium* (0.4%), and adenovirus (0.1%) (*45*). Prevalence of >1 pathogen was higher for children <10 years of age (4.6%) compared with children 10–20 years of age (0.6%) and adults >20 years of age (1.2%). *Blastocystis hominis*, which the authors did not consider pathogenic and was not included in the reported 2.6% prevalence, was detected in 6.0% of stool specimens.

Our results indicate substantially higher prevalence of gut pathogens compared with those studies. However, we detected some individual pathogens less frequently than in other similar studies in the United States. Among infants in Denver, Colorado, USA, in 1990, an estimated 16% of those attending daycare and 9% of those not enrolled had *Giardia duodenalis* detected in stool specimens (*46*). In 1991, the prevalence of *Cryptosporidium* was 3% and *G. duodenalis* 7% among children attending daycare centers in Fulton County, Georgia, USA (*47*). Those values are higher than the 0.4% (2/488) prevalence we observed for *Giardia* and the 0% prevalence for *Cryptosporidium*, although the Colorado and Georgia studies took place more than 30 years ago in different settings and populations. More recently, Tisdale et al. (*48*) used the TAC platform to screen adults traveling internationally from the United States and Germany to low- and middle-income countries for 22 pathogens. Similar to our results, they detected >1 pathogen in stool specimens from 21% of asymptomatic controls.

One limitation of this study is that logistical constraints did not enable analysis of fresh specimens. Transport and storage conditions (time, temperature, and transport media) can influence recovery of pathogen-associated nucleic acids, potentially lowering the sensitivity of molecular assays we used and possibly leading to false-negative results if DNA or RNA fell below our detection limits. Although we attempted to reduce time-to-analysis and to optimize storage conditions to preserve the stability of DNA and RNA, some loss of signal is unavoidable. We assessed Zn-PVA’s performance in preserving nucleic acids in spiked controls (Appendix). In addition, we had missing data in our surveys because of logistical difficulties imposed by the COVID-19 pandemic, such as the need for participants to complete surveys at home and mail them separately from specimens. In addition, some missing data may have been the result of hesitancy to share sanitary conditions because straight pipe discharge of domestic wastewater (*8*) is illegal in the study area (*7*). To mitigate the effect of this missing data, we used MICE and obtained similar results by using this imputation approach compared with analysis on the complete dataset. Further, we were unable to conduct household visits to confirm water and sanitation infrastructure characteristics, including those that may be additional important risk factors for exposure to key pathogens, including wastewater discharges, water source characteristics, soil types, and other environmental variables.

In conclusion, our results suggest that children in households in this region that are reliant on domestic wells may experience increased risks for enteric pathogen exposure compared with children in households with water supplied by utilities. Elevated levels of fecal contamination in groundwater (*12*) could be related to documented deficiencies in rural sanitation in the region, and water as a proximal exposure pathway merits further exploration. New models for infrastructure delivery and management may help expand services, given the limitations of the current paradigm of each household being fully responsible for waste management despite the potential for collective impacts on public health.

Appendix Additional information about risk factors for enteric pathogen exposure among children in Black Belt Region of Alabama, USA.
