# Supplementary material for: Risk Factors for Enteric Pathogen Exposure among Children in Black Belt Region of Alabama, USA
Source: Emerg Infect Dis. 2023 Dec;29(12):2433–41. doi: 10.3201/eid2912.230780 (PMC10683812; doi:10.3201/eid2912.230780)
Supplement: Appendix — Additional information about risk factors for enteric pathogen exposure among children in Black Belt Region of Alabama, USA. [file 23-0780-Techapp-s1.pdf]

Article DOI: https://doi.org/10.3201/eid2912.230780

# Risk Factors for Enteric Pathogen Exposure among Children in Black Belt Region of Alabama, USA

## Appendix

### Zn-PVA Validation

The recovery of *Giardia duodenalis* and *Shigella sonnei* from stool were assessed using different preservative conditions over a period of 8 weeks. First, canine stools collected form a local shelter. Then, an aliquot of each sample was mixed 1:1 into five preservation buffers, which included Zn-PVA (ProtocolTM Parasitology System, Thermo Scientific, Middletown, VA), Total-FixTM (Medical Chemical Corp, Torrance, CA), Universal Extraction (UNEX) buffer (*1*), Nucleic Acid Preservation (NAP) buffer (*2*), and 70% ethanol (Fisher Scientific, Hampton, NH). During mixing, we spiked each aliquot with $\approx 10^6$ *Giardia duodenalis* cysts and $10^8$ *Shigella sonnei* cells (BEI Resources, Manassas, VA). Stool preservative mixtures were stored at ambient temperatures, except Zn-PVA which we assessed at ambient and at 4°C because samples were shipped at ambient conditions but stored at 4°C in the lab. Nucleic acids were extracted from the aliquots using the same protocol as for children's stools immediately upon aliquot preparation and then intermittently over a period of 8 weeks. Finally, gene targets for the two pathogens were quantified using digital PCR (dPCR) to determine the temporal reduction in DNA recovery.

The two PCR assays used were adapted and optimized for dPCR using *Giardia duodenalis* (*3*) and *Shigella sonnei* (*4*) assays published for real-time PCR. Assays were validated and optimized using the QIAcuity Four Digital PCR system (QIAcuity 4, Qiagen, Hilden, Germany). Positive control materials were custom gBlocks (IDT, Coralville, IA) containing each assay's target sequence. PCR reactions were made by combining 2 μL of template with 38 μL of mastermix (Probe PCR Master Mix, Qiagen, Hilden, Germany) and run using 26k 24-well Nanoplates (Qiagen, Hilden, Germany). The Thermocycling conditions used

were 95°C for 2 minutes, followed by 40 cycles of 95°C for 15 seconds and 60°C for 1 minute. Partition fluorescence was measured using preset imaging settings in relative fluorescence units (RFU). Six negative process controls (preservative only) were extracted corresponding to each preservative on days 0 and 28, and from one negative extraction control (water) on each extraction day. One negative PCR control (water) and one positive control was run on each dPCR plate. All negative controls tested negative. Extracts were stored at −80°C until analysis. Thresholding was performed manually by selecting the mid-point between the positive and negative bands in the QIAcuity Software Suite (Qiagen, Hilden, Germany).

Data analysis was performed in Excel (Microsoft, Seattle, Washington) to convert gene copies per µL into gene copies per gram of stool and calculate the mean $\log_{10}$ gene copies and differences in those values over time.

## Results

We observed heterogenous results for the decay of *Giardia* and *Shigella* DNA in the five preservation buffers (Appendix Table 2, Appendix Figure 3). For recovery of DNA from Giardia cysts, UNEX performed best, followed by ZnPVA at 4°C. Whereas for the recovery of DNA from Shigella cells, NAP performed best, followed by UNEX. For both pathogens ZnPVA at 4°C outperformed ZnPVA at ambient conditions. There was typically a 2-week gap from sample collection to receipt at the lab (median = 14 days, IQR = 11, 21) and DNA was extracted approximately 2 weeks later (median = 15 days, IQR = 8, 28). For a hypothetical sample stored at ambient for 14 days and at 4°C for 15 days, this suggests a 0.53 $\log_{10}$ decrease in the *Giardia* concentration and a 0.55 $\log_{10}$ decrease in the *Shigella* concentration would have occurred.

### References

1. Hill VR, Narayanan J, Gallen RR, Ferdinand KL, Cromeans T, Vinjé J. Development of a nucleic acid extraction procedure for simultaneous recovery of DNA and RNA from diverse microbes in water. Pathogens. 2015;4:335–54. PubMed https://doi.org/10.3390/pathogens4020335

2. Camacho-Sanchez M, Burraco P, Gomez-Mestre I, Leonard JA. Preservation of RNA and DNA from mammal samples under field conditions. Mol Ecol Resour. 2013;13:663–73. PubMed https://doi.org/10.1111/1755-0998.12108

3. Liu J, Gratz J, Amour C, Nshama R, Walongo T, Maro A, et al. Optimization of quantitative PCR methods for enteropathogen detection. PLoS One. 2016;11:e0158199. PubMed https://doi.org/10.1371/journal.pone.0158199

4. Lin WS, Cheng CM, Van KT. A quantitative PCR assay for rapid detection of *Shigella* species in fresh produce. J Food Prot. 2010;73:221–33. PubMed https://doi.org/10.4315/0362-028X-73.2.221

5. Qvarnstrom Y, Visvesvara GS, Sriram R, da Silva AJ. Multiplex real-time PCR assay for simultaneous detection of *Acanthamoeba* spp., *Balamuthia mandrillaris*, and *Naegleria fowleri.* J Clin Microbiol. 2006;44:3589–95. PubMed https://doi.org/10.1128/JCM.00875-06

6. Sow D, Parola P, Sylla K, Ndiaye M, Delaunay P, Halfon P, et al. Performance of real-time polymerase chain reaction assays for the detection of 20 gastrointestinal parasites in clinical samples from Senegal. Am J Trop Med Hyg. 2017;97:173–82. PubMed https://doi.org/10.4269/ajtmh.16-0781

7. Rudko SP, Ruecker NJ, Ashbolt NJ, Neumann NF, Hanington PC. *Enterobius vermicularis* as a novel surrogate for the presence of helminth ova in tertiary wastewater treatment plants. Appl Environ Microbiol. 2017;83:e00547–17. PubMed https://doi.org/10.1128/AEM.00547-17

8. Costafreda MI, Bosch A, Pintó RM. Development, evaluation, and standardization of a real-time TaqMan reverse transcription-PCR assay for quantification of hepatitis A virus in clinical and shellfish samples. Appl Environ Microbiol. 2006;72:3846–55. PubMed https://doi.org/10.1128/AEM.02660-05

9. Perchetti GA, Nalla AK, Huang ML, Zhu H, Wei Y, Stensland L, et al. Validation of SARS-CoV-2 detection across multiple specimen types. J Clin Virol. 2020;128:104438. PubMed https://doi.org/10.1016/j.jcv.2020.104438

10. Stokdyk JP, Firnstahl AD, Spencer SK, Burch TR, Borchardt MA. Determining the 95% limit of detection for waterborne pathogen analyses from primary concentration to qPCR. Water Res. 2016;96:105–13. PubMed https://doi.org/10.1016/j.watres.2016.03.026

11. Kaarme J, Hickman RA, Nevéus T, Blomberg J, Öhrmalm C. Reassuringly low carriage of enteropathogens among healthy Swedish children in day care centres. Public Health. 2016;140:221–7. PubMed https://doi.org/10.1016/j.puhe.2016.05.011

**Appendix Table 1.** TAC performance

| Target | Target Gene | y-intercept | $R^2$ | Efficiency | 95% limit of detection † | Reference |
|---|---|---|---|---|---|---|
| enteric 16S | 16S | 38.9 | 0.998 | 101% | 0.60 | (*3*) |
| *Acanthamoeba* spp. | 18S rRNA | 37.8 | 1.000 | 97% | 23 | (*5*) |
| Adenovirus 40/41* | Fiber gene | NA | 0.670 | NA | NA | (*3*) |
| astrovirus | Capsid | 37.5 | 0.998 | 87% | 6.2 | (*3*) |
| *Balantidium coli* | ITS-1 | 37.9 | 1.000 | 97% | 2.2 | (*6*) |
| *Blastocystis* spp. | 18S rRNA | 40.6 | 0.997 | 100% | 2.2 | (*3*) |
| *Cystoisospora belli* | 18S rRNA | 37.8 | 0.999 | 99% | 6.2 | (*3*) |
| *Cyclospora cayetanensi* | 18S rRNA | 37.2 | 0.998 | 99% | 2.2 | (*3*) |
| *Campylobacter jejuni/coli* | *cadF* | 38.3 | 0.999 | 99% | 21 | (*3*) |
| *Clostridioides difficile* | *tcdB* | 37.5 | 0.999 | 96% | 6.2 | (*3*) |
| *Cryptosporidium* spp. | 18S rRNA | 38.0 | 0.999 | 97% | 0.6 | (*3*) |
| DNA control (phocine herpes virus) | *gB* | 37.0 | 0.998 | 100% | 6.2 | (*3*) |
| *Enterocytozoon bieneusi* | ITS | 37.2 | 0.999 | 102% | 4.8 | (*3*) |
| *E. coli* O157:H7 | *rfbE* | 38.0 | 1.000 | 95% | 2.2 | (*3*) |
| *Encephalitozoon intestinalis* | SSU rRNA | 38.5 | 0.999 | 98% | 2.2 | (*3*) |
| *Enterobius vermicularis* | 5S | 38.6 | 0.999 | 95% | 72 | (*7*) |
| EAEC (aaiC) | *aaiC* | 38.2 | 0.999 | 96% | 6.2 | (*3*) |
| EAEC (aatA) | *aatA* | 37.7 | 0.998 | 96% | 23 | (*3*) |
| *Entamoeba hystolytica* | 18S rRNA | 38.0 | 0.996 | 102% | 6.2 | (*3*) |
| *Entamoeba* spp. | 18S rRNA | 37.3 | 0.974 | 104% | 21 | (*3*) |
| EPEC (typical) | *bfpA* | 37.5 | 0.999 | 98% | 6.2 | (*3*) |
| EPEC (atypical) | *eae* | 37.6 | 0.999 | 98% | 2.2 | (*3*) |
| ETEC (LT) | *LT* | 47.6 | 0.990 | 94% | 291 | (*3*) |
| ETEC (STh) | *STh* | 38.8 | 0.999 | 98% | 6.2 | (*3*) |
| ETEC (STp) | *STp* | 37.3 | 0.999 | 99% | 2.2 | (*3*) |
| *Giardia* spp. | 18S rRNA | 37.9 | 1.000 | 96% | 6.2 | (*3*) |
| *Helicobacter pylori* | *ureC* | 37.7 | 0.998 | 97% | 6.2 | (*3*) |
| hepatitis A virus* | NCR | NA | 0.840 | 132% | NA | (*8*) |
| *Shigella*/EIEC | *ipaH* | 37.5 | 0.999 | 99% | 23 | (*3*) |
| MS2 (RNA control) | *MS2g1* | 37.5 | 0.999 | 90% | 1.0 | (*3*) |
| Norovirus GII | ORF1–2 | 37.0 | 0.999 | 92% | 23 | (*3*) |
| Norovirus GI | ORF1–2 | 35.9 | 0.997 | 93% | 23 | (*3*) |
| *Plesiomonas shigelloides* | *gyrB* | 38.2 | 1.000 | 96% | 23 | (*3*) |
| rotavirus | NSP3 | 38.0 | 0.998 | 91% | 6.2 | (*3*) |
| *Salmonella* spp. | *invA* | 38.4 | 1.000 | 96% | 2.2 | (*3*) |
| Sapovirus I/II/IV | RdRp | 38.2 | 0.998 | 88% | 2.2 | (*3*) |
| Sapovirus V | RdRp | 36.7 | 0.999 | 91% | 2.2 | (*3*) |
| SARS-CoV-2 | N1 | 36.2 | 0.995 | 92% | 6.2 | (*9*) |
| STEC (stx1) | *stx1* | 39.9 | 1.000 | 97% | 72 | (*3*) |
| STEC (stx2) | *stx2* | 38.3 | 0.967 | 98% | 96 | (*3*) |
| *Yersinia enterocolitica* | *lytA* | 38.3 | 0.998 | 94% | 2.2 | (*3*) |

*Excluded due to poor standard curve performance
†Stokdyk *et al.* 2016 (*10*); units are gene copies per reaction.

**Appendix Table 2.** MIQE Checklist

| Item to check | Importance | Checklist |
|---|---|---|
| Experimental design | | |
| Definition of experimental and control groups | E | Cross-sectional study with no intervention or control group |
| Number within each group | E | Stools from 488 children were analyzed |
| Assay carried out by core lab or investigator's lab? | D | Investigator's lab |
| Sample | | |
| Description | E | 150 mg of stool preserved 1:1 in ZnPVA (75mg of stool and 75mg of preservative) |
| Volume/mass of sample processed | D | 150 mg |
| Microdissection or macrodissection | E | Not applicable |
| Processing procedure | E | Shipped at ambient, and stored at 4C |
| If frozen - how and how quickly? | E | Not frozen |
| If fixed - with what, how quickly? | E | Preserved in ZnPVA at the time of stool passage |
| Sample storage conditions and duration (especially for FFPE samples) | E | Median 14 d from collection to analysis. Median 15 d from receipt to DNA extraction. |
| Nucleic acid extraction | | |
| Procedure and/or instrumentation | E | See methods section |
| Name of kit and details of any modifications | E | QIAamp 96 Virus QIAcube HT Kit automated on a QIAcube HT |
| Source of additional reagents used | D | Precellys SK38 bead beating tubes (Bertin Technologies, Rockville, MD) |
| Details of DNase or RNase treatment | E | Not applicable |
| Contamination assessment (DNA or RNA) | E | At least one extraction negative control was included during each day of extractions |
| Nucleic acid quantification | E | Qubit 1X HS dsDNA Kit |
| Instrument and method | E | Qubit 4 Fluorometer |
| RNA integrity method/instrument | E | Not measured |
| Inhibition testing (Cq dilutions, spike or other) | E | Monitored amplification of spiked controls |
| REVERSE TRANSCRIPTION | | |
| Complete reaction conditions | E | One-step reverse transcription |
| Amount of RNA and reaction volume | E | Reaction volume = 1.5 µL |
| Priming oligonucleotide (if using GSP) and concentration | E | Proprietary |
| Reverse transcription and concentration | E | ArrayScript Reverse transcription |
| Temperature and time | E | 45°C for 20 min |
| Manufacturer of reagents and catalog numbers | D | Applied Biosystems, AgPath-ID One-Step RT-PCR Reagents, Catalog number: 4387391 |
| qPCR target information | | |
| If multiplex, efficiency and LOD of each assay. | E | Appendix Table 1 |
| Location of amplicon | D | Appendix Table 1 |
| *In silico* specificity screen (BLAST, etc) | E | We BLASTed all assays to confirm specificity before ordering the custom TAC. |
| qPCR oligonucleotides | | |
| Primer sequences | E | Appendix Table 2 |
| Probe sequences | D** | Appendix Table 2 |
| Location and identity of any modifications | E | No modifications |
| Manufacturer of oligonucleotides | D | ThermoFisher Scientific |
| qPCR protocol | | |
| Complete reaction conditions | E | 45°C for 20 min and 95°C for 10 min, followed by 45 cycles of 95°C for 15 s and 60°C for 1 min |
| Reaction volume and amount of cDNA/DNA | E | 40 µL of template with 60 µL of AgPath-ID One-Step RT-PCR Reagents |
| Primer, (probe), Mg++ and dNTP concentrations | E | All assays contained the same concentrations of primers (900 nmol/L) and probe (250 nmol/L). The Mg2+ and dNTP concentrations are not listed in the in the User Guide. |
| Polymerase identity and concentration | E | AmpliTaq Gold polymerase |
| Buffer/kit identity and manufacturer | E | AgPath-ID One-Step RT-PCR Reagents |
| Additives (SYBR Green I, DMSO, etc.) | E | No additives |
| Manufacturer of plates/tubes and catalog number | D | ThermoFisher Scientific |
| Complete thermocycling parameters | E | 45°C for 20 min and 95°C for 10 min, followed by 45 cycles of 95°C for 15 s and 60°C for 1 min |
| Reaction setup (manual/robotic) | D | Manual set-up in a disinfected dead air box (10% bleach with fifteen minutes of contact time, UV for fifteen minutes, and a final cleaning step with 70% ethanol) |
| Manufacturer of qPCR instrument | E | ThermoFisher Scientfic |

| Item to check | Importance | Checklist |
|---|---|---|
| qPCR validation | | |
| Evidence of optimisation (from gradients) | D | See Liu *et al*. 2016 (*3*) |
| Specificity (gel, sequence, melt, or digest) | E | See Liu *et al*. 2016 (*3*) |
| Standard curves with slope and y-intercept | E | Appendix Table 1 |
| PCR efficiency calculated from slope | E | Appendix Table 1 |
| r2 of standard curve | E | Appendix Table 1 |
| Evidence for limit of detection | E | Appendix Table 1 |
| Data analysis | | |
| qPCR analysis program (source, version) | E | QuantStudio Real-Time PCR Software V1.2 CDC |
| Cq method determination | E | Manual thresholding |
| Results of NTCs | E | We observed no amplification before at Ct of 40 in our two PCR negative controls. Among the 12 negative extraction controls, we observed no amplification before a Ct of 40. |
| Justification of number and choice of reference genes | E | |
| Description of normalization method | E | Normalized to mass of stool ZnPVA mixture extracted from (150mg) |
| Number and concordance of biologic replicates | D | See results section. |
| Number and stage (RT or qPCR) of technical replicates | E | See results section. |
| Statistical methods for result significance | E | See methods section |
| Software (source, version) | E | R Studio V2.2.2 |

**Appendix Table 3.** Primer and probe sequences

| Pathogen | Primer or probe sequence (5′ - 3′) |
|---|---|
| Astrovirus | Fwd: CAGTTGCTTGCTGCGTTCA<br>Rev: CTTGCTAGCCATCACACTTCT<br>Probe: CACAGAAGAGCAACTCCATCGC |
| Norovirus GI | Fwd: CGYTGGATGCGNTTYCATGA<br>Rev: CTTAGACGCCATCATCATTYAC<br>Probe: TGGACAGGAGATCGC |
| Norovirus GII | Fwd: CARGARBCNATGTTYAGRTGGATGAG<br>Rev: TCGACGCCATCTTCATTCACA<br>Probe: TGGGAGGGCGATCGCAATCT |
| Sapovirus (I, II, IV) | Fwd: GAYCAGGCTCTCGCYACCTAC<br>Rev: CCCTCCATYTCAAACACTA<br>Probe: CYTGGTTCATAGGTGGTRCAG |
| Sapovirus V | Fwd: TTTGAACAAGCTGTGGCATGCTAC<br>Rev: CCCTCCATYTCAAACACTA<br>Probe: CAGCTGGTACATTGGTGGCAC |
| Adenovirus 40/41 | Fwd: AACTTTCTCTCTTAATAGACGCC<br>Rev: AGGGGGCTAGAAAACAAAA<br>Probe: CTGACACGGGCACTCT |
| Rotavirus | Fwd: ACCATCTWCACRTRACCCTCTATGAG<br>Rev: GGTCACATAACGCCCCTATAGC<br>Probe: AGTTAAAAGCTAACACTGTCAAA |
| *Campylobacter jejuni* or *coli* | Fwd: CTGCTAAACCATAGAAATAAAATTTCTCAC<br>Rev: CTTTGAAGGTAATTTAGATATGGATAATCG<br>Probe: CATTTTGACGATTTTTGGCTTGA |
| *C. difficile* | Fwd: GGTATTACCTAATGCTCCAAATAG<br>Rev: TTTGTGCCATCATTTTCTAAGC<br>Probe: CCTGGTGTCCATCCTGTTTC |
| EAEC (aaiC) | Fwd: ATTGTCCTCAGGCATTTCAC<br>Rev: ACGACACCCCTGATAAACAA<br>Probe: TAGTGCATACTCATCATTTAAG |
| EAEC (aatA) | Fwd: CTGGCGAAAGACTGTATCAT<br>Rev: TTTTGCTTCATAAGCCGATAGA<br>Probe: TGGTTCTCATCTATTACAGACAGC |
| STEC (stx1) | Fwd: ACTTCTCGACTGCAAAGACGTATG<br>Rev: ACAAATTATCCCCTGWGCCACTATC<br>Probe: CTCTGCAATAGGTACTCC |
| STEC (stx2) | Fwd: CCACATCGGTGTCTGTTATTAACC<br>Rev: GGTCAAAACGCGCCTGATAG<br>Probe TTGCTGTGGATATACGAGG |

| Pathogen | Primer or probe sequence (5′ - 3′) |
|---|---|
| EPEC (eae) | Fwd: CATTGATCAGGATTTTTCTGGTGATA |
| | Rev: CTCATGCGGAAATAGCCGTTA |
| | Probe: ATACTGGCGAGACTATTTCAA |
| EPEC (bfpA) | Fwd: TGGTGCTTGCGCTTGCT |
| | Rev: CGTTGCGCTCATTACTTCTG |
| | Probe: CAGTCTGCGTCTGATTCCAA |
| ETEC LT | Fwd: TTCCCACCGGATCACCAA |
| | Rev: CAACCTTGTGGTGCATGATGA |
| | Probe: CTTGGAGAGAAGAACCCT |
| ETEC ST | Fwd h: GCTAAACCAGYAGRGTCTTCAAAA |
| | Fwd p: TGAATCACTTGACTCTTCAAAA |
| | Rev h: CCCGGTACARGCAGGATTACAACA |
| | Rev p: GGCAGGATTACAACAAAGTT |
| | Probe h: TGGTCCTGAAAGCATGAA |
| | Probe p: TGAACAACACATTTTACTGCT |
| EIEC or *Shigella* | Fwd: CCTTTTCCGCGTTCCTTGA |
| | Rev: CGGAATCCGGAGGTATTGC |
| | Probe: CGCCTTTCCGATACCGTCTCTGCA |
| *Salmonella* | Fwd: CTCACCAGGAGATTACAACATGG |
| | Rev: AGCTCAGACCAAAAGTGACCATC |
| | Probe: CACCGACGGCGAGACCGACTTT |
| *E. coli* O157 | Fwd: TTTCACACTTATTGGATGGTCTCAA |
| | Rev: CGATGAGTTTATCTGCAAGGTGAT |
| | Probe: CTCTCTTTCCTCTGCGGTCCT |
| *Cryptosporidium* | Fwd: GGGTTGTATTTATTAGATAAAGAACCA |
| | Rev: AGGCCAATACCCTACCGTCT |
| | Probe: TGACATATCATTCAAGTTTCTGAC |
| *Giardia* spp. | Fwd: GACGGCTCAGGACAACGGTT |
| | Rev: TTGCCAGCGGTGTCCG |
| | Probe: CCCGCGGCGGTCCCTGCTAG |
| *E. histolytica* | Fwd: ATTGTCGTGGCATCCTAACTCA |
| | Rev: GCGGACGGCTCATTATAACA |
| | Probe: TCATTGAATGAATTGGCCATTT |
| *Entamoeba* spp. | Fwd: AAACGATGTCAACCAAGGATTG |
| | Rev: TCCCCCTGAAGTCCATAAACTC |
| | Probe: CCTTGTTCAGAACTTAAAGAGAAA |
| *Blastocystis* spp. | Fwd: TGGTCCGRTGAACACTTTGGAT |
| | Rev: CCTACGGAAACCTTGTTACGACTTCA |
| | Probe: CTTCCTCTAAATGRTAAGATT |
| 16s | Fwd: TGCAAGTCGAACGAAGCACTTTA |
| | Rev: GCAGGTTACCCACGCGTTAC |
| | Probe: CGCCACTCAGTCACAAA |
| PhHV | Fwd: GGGCGAATCACAGATTGAATC |
| | Rev: GCGGTTCCAAACGTACCAA |
| | Probe: TATGTGTCCGCCACCATCT |
| *Yersinia enterocolitica* | Fwd: TGATTCACCAGCAGCAATAC |
| | Rev: GGCATCATGAAAGGCGG |
| | Probe: TGTCGGTTTCTCCTTCCAGG |
| *Heliobacter pylori* | Fwd: GACACCAGAAAAAGCGGCTA |
| | Rev: AGCGCATGTCTTCGGTTAAA |
| | Probe: TCACTAAAGCGTTTTCTACC |
| *Plesiomonas shigelloides* | Fwd: CCGCCGTGAAGGCAAAG |
| | Rev: GCTACCGGCTCACCCAGAT |
| | Probe: CACACCCAAGAATAC |
| *Cyclospora cayetanensi* | Fwd: AAAAGCTCGTAGTTGGATTTCTG |
| | Rev: AACACCAACGCACGCAGC |
| | Probe: AAGGCCGGATGACCACGA |
| *Cystoisospora belli* | Fwd: ATATTCCCTGCAGCATGTCTGTTT |
| | Rev: CCACACGCGTATTCCAGAGA |
| | Probe: CAAGTTCTGCTCACGCGCTTCTGG |
| *Blastocystis* spp. | Fwd: TGGTCCGRTGAACACTTTGGAT |
| | Rev: CCTACGGAAACCTTGTTACGACTTCA |
| | Probe: CTTCCTCTAAATGRTAAGATT |
| *Enterocytozoon bieneusi* | Fwd: TGTGTAGGCGTGAGAGTGTATCTG |
| | Rev: CATCCAACCATCACGTACCAATC |
| | Probe: CACTGCACCCACATCCCTCACCCTT |
| *Encephalitozoon intestinalis* | Fwd: CACCAGGTTGATTCTGCCTGAC |
| | Rev: CTAGTTAGGCCATTACCCTAACTACCA |
| | Probe: CTATCACTGAGCCGTCC |

| Pathogen | Primer or probe sequence (5′ - 3′) |
|---|---|
| *Balantidium coli* | Fwd: TGCAATGTGAATTGCAGAACC |
| | Rev: TGGTTACGCACACTGAAACAA |
| | Probe: CTGGTTTAGCCAGTGCCAGTTGC |
| *Acanthamoeba* spp. | Fwd: CCCAGATCGTTTACCGTGAA |
| | Rev: TAAATATTAATGCCCCCAACTATC |
| | Probe: CTGCCACCGAATACATTAGCATGG |
| Hepatitis A Virus | Fwd: TCACCGCCGTTTGCCTAG |
| | Rev: GGAGAGCCCTGGAAGAAAG |
| | Probe: TTAATTCCTGCAGGTTCAGG |
| SARS-CoV-2 | Fwd: GACCCCAAAATCAGCGAAAT |
| | Rev: TCTGGTTACTGCCAGTTGAATCTG |
| | Probe: ACCCCGCATTACGTTTGGTGGACC |

**Appendix Table 4.** Risk factors for ≥1 pathogen detection (using only complete cases, n = 341)

| Variable | Reference | Exposure | RR (95% CI) | aRR (95% CI) |
|---|---|---|---|---|
| Pay a water bill | Yes | No | 1.8 (1.3, 2.6) | 1.8 (1.3, 2.6) |
| Sanitation | Sewer connection | Cesspit | NA | NA |
| | | Other | NA | NA |
| | | Septic Tank | 0.90 (0.59, 1.4) | 0.91 (0.60, 1.4) |
| | | Straight Pipe | 0.98 (0.53, 1.8) | 0.91 (0.49, 1.7) |
| Child's Screen Time | <2 h | 2–4 h | 0.66 (0.42, 1.0) | 0.71 (0.45, 1.1) |
| | | >4 h | 0.67 (0.43, 1.0) | 0.64 (0.41, 1.0) |
| Gender | Male | Female | 0.91 (0.66, 1.3) | 0.92 (0.66, 1.3) |
| International Travel | No | Yes | 0.92 (0.34, 2.5) | 1.0 (0.37, 2.9) |
| Raw Sewage | No | Yes | 1.2 (0.65, 2.3) | 1.2 (0.70, 2.1) |
| Age | <5 y | 5–10 y | 0.77 (0.39, 1.5) | 1.0 (0.48, 2.1) |
| | | >10 y | 0.88 (0.46, 1.7) | 1.1 (0.55, 2.4) |

**Appendix Table 5.** Decay constants for different preservation buffers

| Target | Preservative | Log10 decay in DNA concentration per day |
|---|---|---|
| *Giardia* | Zn PVA (4C) | −0.0037 |
| *Giardia* | Zn PVA (20C) | −0.034 |
| *Giardia* | UNEX | −0.0008 |
| *Giardia* | TotalFix | −0.0541 |
| *Giardia* | NAP | −0.0358 |
| *Giardia* | 70% Ethanol | −0.0469 |
| *Shigella* | Zn PVA (4C) | −0.0085 |
| *Shigella* | Zn PVA (20C) | −0.0303 |
| *Shigella* | UNEX | −0.003 |
| *Shigella* | TotalFix | −0.0154 |
| *Shigella* | NAP | −0.0003 |
| *Shigella* | 70% Ethanol | −0.0442 |

**Appendix Table 6.** Comparison with Swedish Children

| Type | Pathogen | Prevalence in rural Alabama | Prevalence among Swedish Children in Daycare (*11*) |
|---|---|---|---|
| Any | ≥1 Pathogen detected | 26% (127/488) | |
| Bacteria | *Clostridioides difficile* (toxin B) | 6.6% (32/488) | 2.5% (11/438) |
| | EPEC (atypical) | 6.1% (30/488) | Not assessed |
| | EAEC | 3.9% (19/488) | Not assessed |
| | *Helicobacter pylori* | 2.3% (11/488) | Not assessed |
| | EPEC (typical) | 1.4% (7/488) | Not assessed |
| | *Yersinia enterocolitica* | 1.0% (5/488) | 0% (0/438) |
| | *E. coli* O157:H7 | 0.8% (4/488) | 0% (0/438) |
| | *Plesiomonas shigelloides* | 0.4% (2/488) | Not assessed |
| | ETEC | 0.4% (2/488) | 1.4% (6/438) |
| | *Shigella*/EIEC | 0.2% (1/488) | 0% (0/438) |
| | *Salmonella* spp. | 0.2% (1/488) | 0% (0/438) |
| | STEC | 0.2% (1/488) | 0% (0/438) |
| | *Campylobacter jejuni/coli* | 0% (0/488) | 0.7% (3/438) |
| Fungus/Algae | *Blastocystis* spp. | 3.7% (18/488) | Not assessed |

| Type | Pathogen | Prevalence in rural Alabama | Prevalence among Swedish Children in Daycare (*11*) |
| --- | --- | --- | --- |
| | *Enterocytozoon bieneusi* | 0% (0/488) | Not assessed |
| | Encephalitozoon intestinalis | 0% (0/488) | Not assessed |
| Protozoa | *Balantidium coli* | 0.6% (3/488) | Not assessed |
| | *Acanthamoeba* spp. | 0.4% (2/488) | Not assessed |
| | *Giardia* spp. | 0.4% (2/488) | 0% (0/438) |
| | *Entamoeba hystolytica* | 0.2% (1/488) | 0% (0/438) |
| | *Cystoisospora belli* | 0% (0/488) | Not assessed |
| | *Cyclospora cayetanensi* | 0% (0/488) | Not assessed |
| | *Cryptosporidium* spp. | 0% (0/488) | 0% (0/438) |
| | *Entamoeba* spp. | 0% (0/488) | Not assessed |
| Virus | norovirus GI/GII | 1.4% (7/488) | 0.7% (3/438) |
| | SARS-CoV-2 | 0.6% (3/488) | Not assessed |
| | rotavirus | 0.4% (2/488) | 0% (0/438) |
| | sapovirus | 0.4% (2/488) | Not assessed |
| | astrovirus | 0.2% (1/488) | Not assessed |

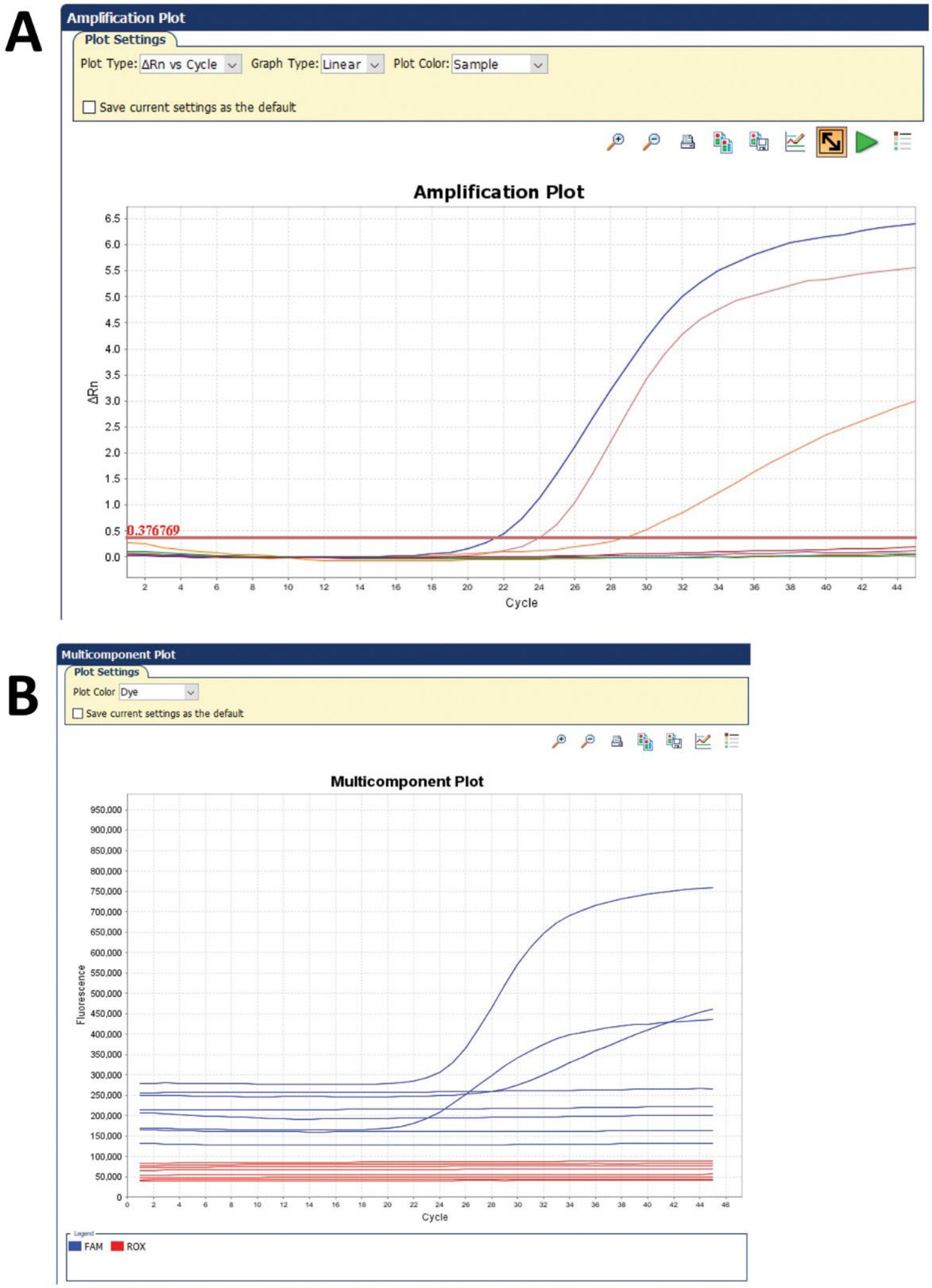


**Appendix Figure 1.** Amplification and multicomponent plots.

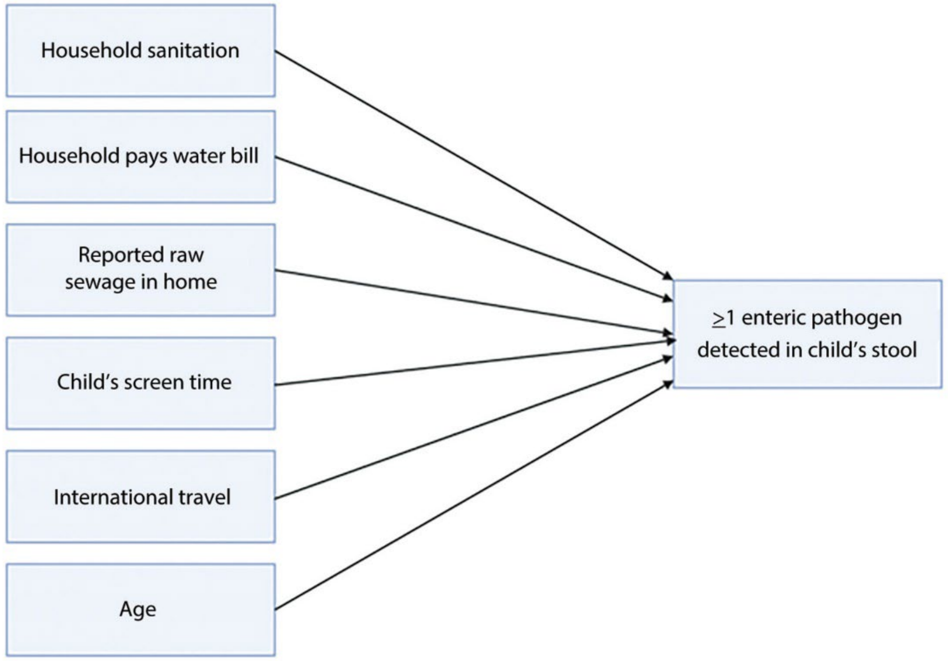


**Appendix Figure 2.** Acyclic graph.

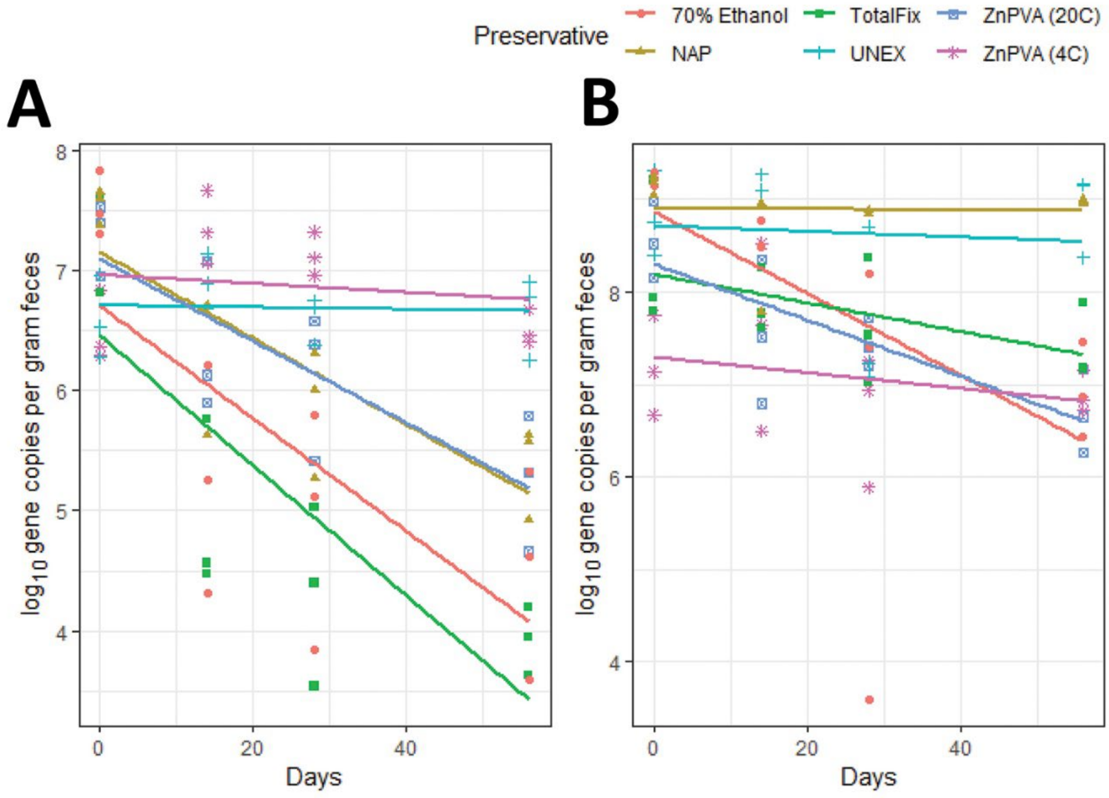


**Appendix Figure 3.** Gene copy recovery.

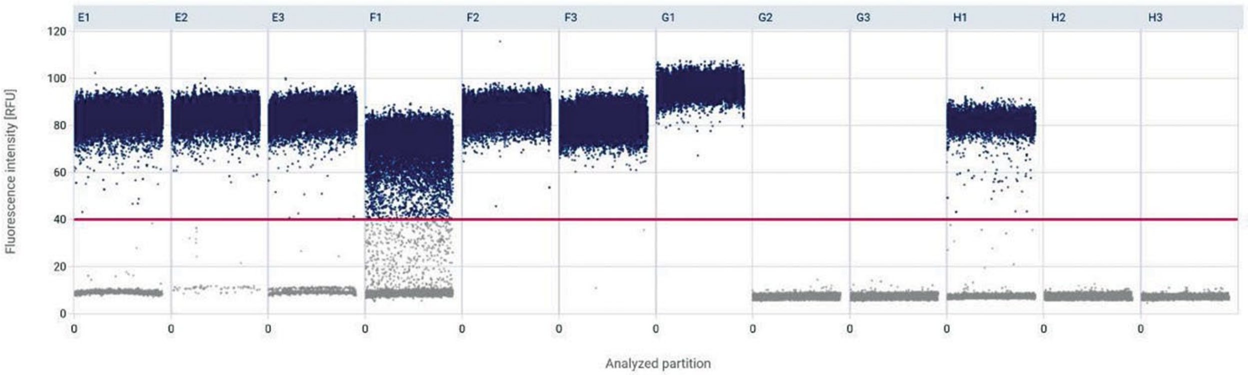


**Appendix Figure 4.** dPCR 2-D Scatterplot. Wells G2, G3, H2, and H3 were negative extraction controls, well H1 was a PCR positive control; all other wells were samples. Samples that were outside the range of quantification (i.e., F2, F3, and G1) were rerun at a 1:10 dilution.
